# Hybrid Hydrogels Augmented via Additive Network Integration (HANI) for Meniscal Tissue Engineering Applications

**DOI:** 10.3390/gels11040223

**Published:** 2025-03-21

**Authors:** Anthony El Kommos, Praveen Magesh, Samantha Lattanze, Andrew Perros, Fotios Andreopoulos, Francesco Travascio, Alicia Jackson

**Affiliations:** 1 Department of Biomedical Engineering, University of Miami, Coral Gables, FL 33146, USA; axe627@miami.edu (A.E.K.); sll8997@miami.edu (S.L.); amp755@miami.edu (A.P.); 2 Department of Mechanical and Aerospace Engineering, University of Miami, Coral Gables, FL 33146, USA; pxm677@miami.edu (P.M.); f.travascio@miami.edu (F.T.); 3 Department of Surgery, University of Miami, Miami, FL 33136, USA; 4 Department of Orthopaedic Surgery, University of Miami, Miami, FL 33136, USA

**Keywords:** confined compression, hydrogel, stress relaxation, poroviscoelastic, meniscus, tissue engineering

## Abstract

Orthopedic soft tissue injuries, such as those to the fibrocartilaginous meniscus in the knee, present a significant clinical challenge, impacting millions globally and often requiring surgical interventions that fail to fully restore mechanical function. Current bioengineered meniscal replacement options that incorporate synthetic and/or natural scaffolds have limitations in biomechanical performance and biological integration. This study introduces a novel scaffold fabrication approach, termed Hybrid Hydrogels Augmented via Additive Network Integration (HANI) with great potential for meniscal tissue engineering applications. HANI scaffolds combine cross-linked gelatin-based hydrogels with polycaprolactone (PCL) additive networks, created via Fused Deposition Modeling (FDM), to enhance mechanical strength and replicate the anisotropic properties of the meniscus. Custom Stereolithography (SLA)-printed molds ensure precise dimensional control and seamless incorporation of PCL networks within the hydrogel matrix. The mechanical evaluation of HANI scaffolds showed improvements in compressive stiffness, stress relaxation behavior, and load-bearing capacity, especially with circumferential and 3D PCL reinforcements, when compared to hydrogel scaffolds without additive networks. These findings highlight HANI’s potential as a cost-effective, scalable, and tunable scaffold fabrication approach for meniscal tissue engineering applications.

## 1. Introduction

Orthopedic soft tissue injuries are a significant clinical concern, with meniscal injuries alone necessitating over 750,000 surgical procedures annually in the United States [1,2]. As a fibrocartilaginous structure in the knee, the menisci play an essential role in absorbing shock and distributing mechanical loads, thereby maintaining joint stability and protecting cartilage from excessive wear [3]. The multi-layered anisotropic architecture of the menisci convert axial loads into tensile stresses, enabling efficient load distribution [4,5]. Despite its importance, there is no viable tissue-engineered solution to effectively restore meniscal function, leaving patients reliant on suboptimal treatments [3].

Current interventions, including partial or total meniscectomy and suture repair, fail to replicate the native mechanical and poroviscoelastic properties of the meniscus. These shortcomings often result in altered joint mechanics, accelerated cartilage degeneration, and an increased risk of osteoarthritis [6,7]. Synthetic and natural implants also have inherent limitations. Synthetic materials, such as poly-ε-caprolactone (PCL), provide mechanical robustness but lack bioactivity, which hinders cellular adhesion, proliferation, and differentiation. Conversely, natural materials like gelatin offer excellent biocompatibility and biodegradability but lack the mechanical strength required for load-bearing applications, leading to scaffold failure under physiological conditions [4,8,9]. A hybrid solution that leverages the strengths of both material types is urgently needed to address these limitations.

Current challenges in meniscal and cartilage tissue engineering include articular cartilage degeneration due to suboptimal load distribution, rigid scaffolds that fail to replicate native biomechanics, and biologically compatible materials that lack sufficient mechanical strength [10,11,12]. Many fabrication techniques, such as electrospinning, freeze-drying, and bioprinting, offer advantages in biocompatibility and structural complexity but often struggle to balance mechanical integrity and physiological function [10,11,12,13].

Advances in additive manufacturing, particularly 3D printing, have shown promise in fabricating meniscal scaffolds with tailored geometries and mechanical properties [14,15,16,17]. However, 3D-printed PCL neomenisci often exhibit excessive stiffness compared to native tissue, disrupting load distribution and increasing surface friction, which can lead to articular cartilage wear and degeneration. Furthermore, these constructs typically lack sufficient porosity and bioactivity to support cellular integration and tissue remodeling [18,19]. Bioprinting offers a more advanced alternative by incorporating cells and bioactive materials into scaffolds, but it remains prohibitively expensive, time-intensive, and difficult to scale for clinical use [20,21,22]. These drawbacks highlight the need for a cost-effective, adaptable approach to meniscal tissue engineering.

**Figure 1:**
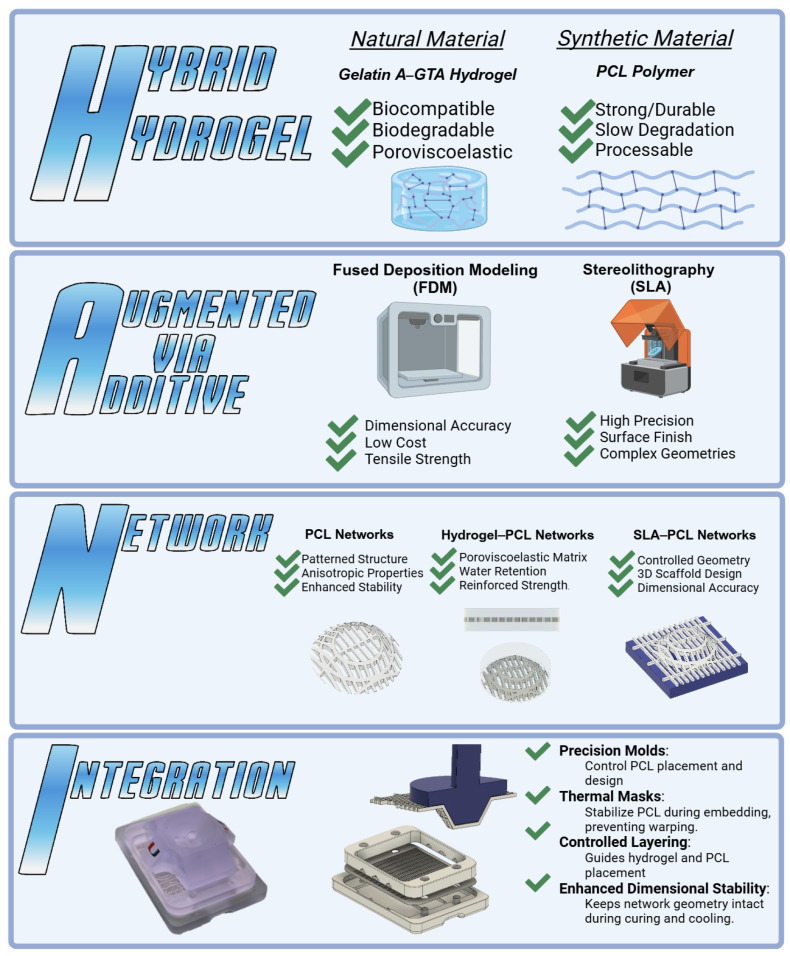
The HANI scaffold combines Hybrid Hydrogel (natural gelatin with synthetic PCL) for balanced biocompatibility and mechanical strength. Augmentation via Additive Manufacturing, FDM, and SLA techniques offer precision in the scaffold design and reinforcement. The Network integrates PCL networks within the hydrogel matrix, mimicking the anisotropic properties of the meniscus. Integration uses precision molding and thermal masks for dimensional stability, ensuring accurate PCL alignment. This approach aims to replicate native meniscal mechanics for effective tissue engineering solutions.

This study presents a new modular scaffold fabrication approach (i.e., Hybrid Hydrogels Augmented via Additive Network Integration (HANI)) to prepare hydrogel constructs with tunable mechanical and biological properties similar to those of meniscal tissue. In this process, hydrogels are fabricated using gelatin (GelA) cross-linked with glutaraldehyde (GTA) to achieve physiologically relevant poroviscoelastic properties and water content. These hydrogels are then augmented with poly-ε-caprolactone (PCL) additive networks, which are fabricated using Fused Deposition Modeling (FDM) to create anisotropic, biomimetic patterns that mimic the structural organization of the native meniscus. Finally, PCL networks are integrated using custom Stereolithography (SLA)-printed molds and thermal masks to ensure precise dimensional accuracy and seamless incorporation into the hydrogel matrix. By integrating tailored PCL networks within the gelatin-based hydrogels, the HANI process (Figure 1) seeks to improve the scaffold’s compressive stiffness and stress relaxation properties while maintaining the inherent benefits of gelatin.

### Application of Stress Relaxation Metrics

Stress relaxation metrics, including peak stress, stress decay, equilibrium stress, the relaxation time constant, and the aggregate modulus, are key indicators of hydrogels’ potential to mimic the mechanical behavior of native meniscal tissue [23,24,25,26]. Hydrogels with higher peak stress values and longer relaxation times are expected to demonstrate greater resistance to mechanical deformation, making them suitable for withstanding dynamic loads such as those encountered during walking or running. These metrics also help determine the HANI’s ability to reduce peak stress transmission to surrounding cartilage, a critical factor in preventing joint degeneration [27,28].

**Figure 2:**
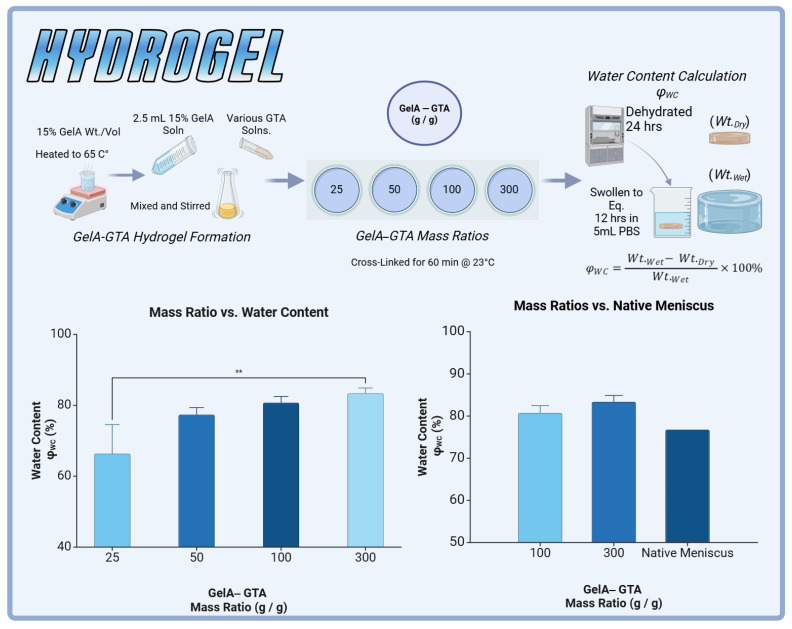
GelA–GTA hydrogel formation and evaluation for meniscal tissue engineering. (**Top panel**) Schematic representation of GelA–GTA hydrogel preparation: 15% GelA solution is heated to 65 °C, mixed with various concentrations of glutaraldehyde (GTA), and cross-linked at 23 °C for 60 min. Hydrogels are dehydrated for 24 h and equilibrated in phosphate-buffered saline (PBS) for 12 h to determine water content ($\phi_{WC}$). (**Bottom left**) Water content (%) increases with higher GelA–GTA mass ratios, with 100:1 and 300:1 formulations achieving values comparable to native meniscus. (**Bottom right**) Comparison of hydrogel water content at 100:1 and 300:1 GelA–GTA mass ratios to native meniscus tissue, demonstrating suitability for mimicking native hydration levels. Statistical significance is denoted by ** *p* < 0.01.

By analyzing hydrogels with gelatin-to-glutaraldehyde (GTA) cross-linking mass ratios of 300 and 100, the impact of cross-linking density and PCL configuration on stress relaxation behavior can be explored (Figure 2). Lower cross-linking concentrations (300 GTA) are expected to exhibit faster stress relaxation and a lower peak modulus, suggesting increased flexibility but reduced mechanical stiffness. In contrast, hydrogels with higher cross-linking (100 GTA) are anticipated to display higher peak stress and peak modulus values, indicating greater stiffness but slower stress relaxation [13]. Moreover, PCL treatment is expected to significantly enhance overall stiffness during compression and equilibrium while still maintaining poroviscoelastic stress relaxation behavior [3,4,5]. These variations highlight the trade-offs between flexibility and stiffness, offering a tunable system to optimize hydrogel properties based on specific clinical needs, such as meniscal repair.

Moreover, the equilibrium modulus provides insight into the material’s ability to maintain its structure under prolonged loads, while the relaxation time constant offers an understanding of how rapidly the hydrogels can redistribute mechanical forces, critical for load-bearing tissues. Together, these parameters inform the design of hydrogel scaffolds that achieve a balance between rapid stress dissipation, important for maintaining joint stability and sustained mechanical support under repetitive loading [27,28].

The versatility of additive manufacturing techniques like SLA and FDM enable precise control over scaffold geometry, cross-linking density, and PCL integration (Figure 1). This adaptability allows the HANI process to produce customizable scaffolds tailored to individual anatomical and mechanical needs, ensuring physiologically relevant dynamic behavior without relying on costly bioprinting methods [29,30]. By maintaining cost-effectiveness and scalability, the HANI approach prioritizes accessibility and clinical applicability while addressing the limitations of current meniscal repair strategies.

The overarching goal is to create orthopedic soft tissue engineered constructs, such as meniscal replacements, that balance mechanical strength, dynamic behavior, and biological compatibility. HANI, a cost-effective and customizable solution, aim to improve clinical outcomes by providing a durable biomimetic scaffold capable of restoring joint functionality and ensuring long-term integration under physiological loads.

## 2. Results and Discussion

This study introduced a novel method of hydrogel augmentation via the HANI method to mimic the native meniscus by leveraging the poroviscoelastic properties of gelatin-based hydrogels and the structural reinforcement of polycaprolactone (PCL). To achieve physiologically relevant properties, various mass ratios of gelatin to glutaraldehyde (GTA) were evaluated for water content, with the goal of approximating the native meniscus, which exhibits a water content of approximately 76% [31]. The 100:1 and 300:1 GelA–GTA mass ratios were selected for further study as they closely matched native meniscal water content, albeit with slightly higher values (Figure 2). This adjustment was intentional, given the plan to incorporate PCL reinforcements, which would increase the overall polymer content and modulate water retention.

Although differences in water content between formulations were not statistically significant, mechanical performance testing revealed substantial variations in load-bearing capacity, compressive stiffness, and stress relaxation behavior. Consequently, the 100:1 and 300:1 gelatin-to-GTA mass ratios were chosen to further assess the scaffolds’ mechanical attributes across various PCL configurations.

The optimization process further involved integrating PCL reinforcement configurations (aligned, circumferential, and 3D) to enhance anisotropic load distribution, energy dissipation, and structural integrity under dynamic physiological forces [32,33]. This synergy between chemical and mechanical properties positions the scaffolds as robust candidates for meniscal tissue engineering, demonstrating their potential to withstand repetitive joint loading while maintaining functionality over time [34].

### 2.1. Peak Stress ($\sigma_{0}$)

**Figure 3:**
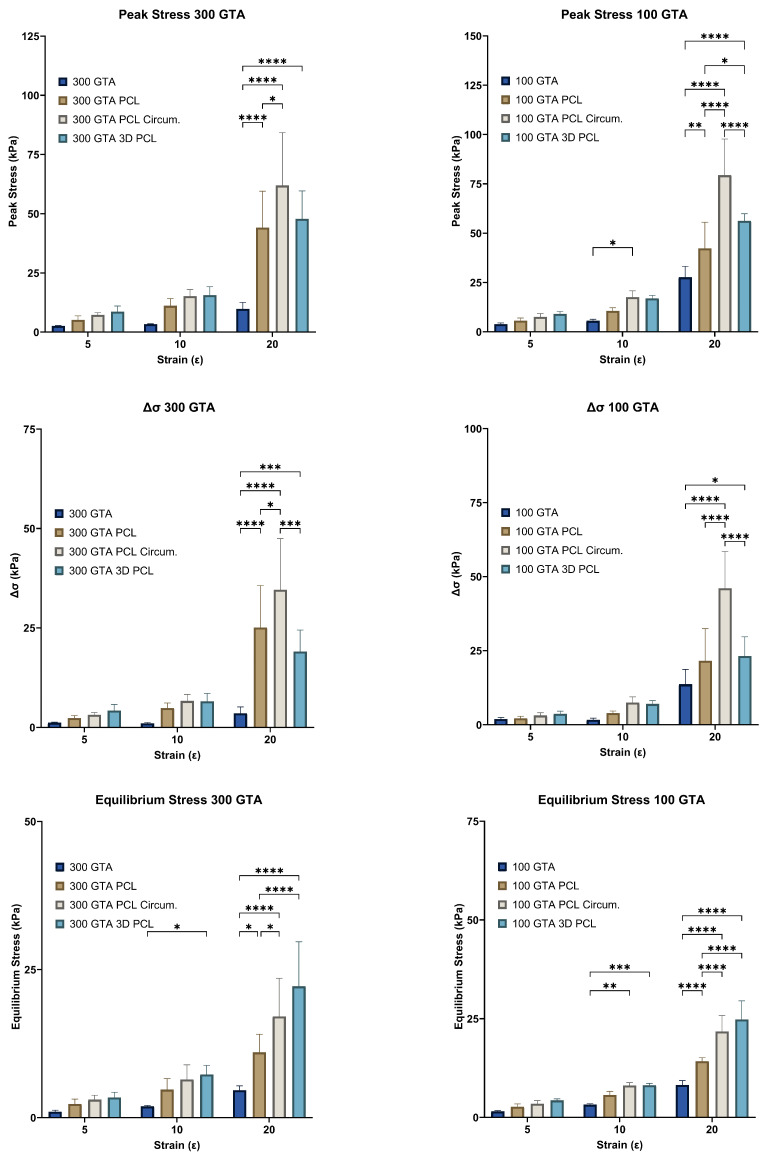
Effect of GelA–GTA mass ratio and PCL reinforcement type on hydrogel mechanical properties across strain levels. Bar graphs display peak stress ($\sigma_{0}$), stress delta (Δσ), and equilibrium stress ($\sigma_{Eq}$) for composite hydrogels with varying GelA–GTA mass ratios (300 and 100) and PCL reinforcement types (no PCL, aligned PCL, circumferential PCL, and 3D PCL) across strain levels of 5%, 10%, and 20%. Significant increases in mechanical properties are observed at higher strain levels, particularly in 100 GTA hydrogels with circumferential and 3D PCL configurations. Statistical significance is denoted by * *p* < 0.05; ** *p* < 0.01; *** *p* < 0.001; and **** *p* < 0.0001.

Peak stress ($\sigma_{0}$), a measure of the maximum stress a material can sustain under load, exhibited substantial improvements with both increased glutaraldehyde (GTA) concentration and polycaprolactone (PCL) reinforcement (Figure 3). For the 300 GTA hydrogels, unreinforced samples displayed modest peak stress values, reaching 2.45 ± 0.20 kPa at a 5% strain and 9.79 ± 2.47 kPa at a 20% strain. Aligned PCL reinforcement more than doubled these values, achieving 5.19 ± 1.50 kPa at a 5% strain and 44.13 ± 13.75 kPa at a 20% strain. Circumferential PCL reinforcement provided even greater improvements, with $\sigma_{0}$ reaching 7.25 ± 0.87 kPa at a 5% strain and 61.90 ± 20.01 kPa at a 20% strain. Similarly, 3D PCL scaffolds enhanced peak stress to 8.03 ± 1.82 kPa and 49.17 ± 13.17 kPa at 5% and 20% strains, respectively.

In the 100 GTA hydrogels, peak stress values were markedly higher than those in the 300 GTA hydrogels for most configurations, except the initial aligned PCL which had slightly lower values at 5% and 20% strains, due to the increased cross-linking density. Unreinforced samples exhibited peak stresses of 3.84 ± 0.60 kPa at a 5% strain and 27.63 ± 4.94 kPa at a 20% strain. Aligned PCL reinforcement further improved $\sigma_{0}$, reaching 5.65 ± 1.22 kPa at a 5% strain and 42.30 ± 11.82 kPa at a 20% strain. Circumferential PCL reinforcement achieved the highest values, with $\sigma_{0}$ increasing to 7.54 ± 1.50 kPa at a 5% strain and 79.38 ± 16.40 kPa at a 20% strain. The 3D PCL configuration also performed well, with peak stresses reaching 9.04 ± 0.11 kPa at a 5% strain and 56.16 ± 2.98 kPa at a 20% strain.

Two-way ANOVA revealed significant effects of strain level and PCL configuration for both GelA–GTA mass ratios on peak stress (*p* < 0.0001). Post hoc Tukey’s tests confirmed that circumferential and 3D PCL configurations significantly outperformed aligned and unreinforced hydrogels (*p* < 0.05). Among the configurations, circumferential PCL reinforcement in the 100 GTA hydrogels yielded the highest peak stress values, highlighting its superior ability to distribute loads effectively.

These results underscore the importance of the HANI’s strategic PCL reinforcement, particularly in the circumferential and 3D configurations, in enhancing the mechanical strength of the hydrogels [35,36,37]. The highest peak stresses observed in the circumferential PCL-reinforced 100 GTA hydrogels mimicked the anisotropic properties of native meniscal tissue, indicating their potential as load-bearing candidates for orthopaedic applications.

### 2.2. Stress Decay (Δσ)

Stress decay (Δσ), a measure of a material’s ability to dissipate stress over time, was significantly influenced by strain level and PCL reinforcement for both GelA–GTA mass ratios (Figure 3). At a 300 GTA ratio, unreinforced hydrogels exhibited low stress decay values across all strain levels, with Δσ reaching only 1.09 ± 0.12 kPa at a 5% strain and 3.55 ± 1.44 kPa at a 20% strain. PCL reinforcement improved stress decay significantly, particularly in the circumferential and 3D configurations. Circumferential PCL reinforcement increased Δσ to 3.18 ± 0.50 kPa at a 5% strain and 34.59 ± 11.51 kPa at a 20% strain, nearly ten folds higher than unreinforced hydrogels. Similarly, the 3D configuration achieved Δσ values of 3.75 ± 1.11 kPa and 20.23 ± 5.75 kPa at 5% and 20% strains, respectively.

In the 100 GTA hydrogels, stress decay values were significantly higher, reflecting the effect of increased cross-linking density. The unreinforced 100 GTA hydrogels demonstrated Δσ values of 1.92 ± 0.49 kPa at a 5% strain and 13.66 ± 4.46 kPa at a 20% strain. With circumferential PCL reinforcement, Δσ reached 3.16 ± 0.83 kPa at a 5% strain and 46.03 ± 11.16 kPa at a 20% strain, the highest value recorded. The 3D PCL configuration also performed well, with Δσ values of 3.93 ± 0.05 kPa and 21.85 ± 3.32 kPa at 5% and 20% strains, respectively. Two-way ANOVA revealed significant effects of strain level and PCL configuration for both GelA–GTA mass ratios on stress decay (*p* < 0.0001), with the circumferential and 3D PCL reinforcements significantly outperforming the aligned and unreinforced hydrogels (*p* < 0.05). These results indicate that strategic reinforcement, particularly circumferential PCL in a 100 GTA matrix, provides superior stress decay performance, enhancing resilience under prolonged compressive loading. This behaviour mimics the viscoelastic properties of native meniscal tissue, making these configurations well-suited for applications requiring durability under repetitive loads [11,38,39,40,41].

### 2.3. Equilibrium Stress ($\sigma_{Eq}$)

Equilibrium stress ($\sigma_{Eq}$), representing the sustained stress level after initial relaxation, improved significantly for both GelA–GTA mass ratios with strain level and PCL reinforcement (Figure 3). For the 300 GTA hydrogels, the unreinforced samples showed low equilibrium stress values, reaching 1.08 ± 0.23 kPa at a 5% strain and 4.62 ± 0.67 kPa at a 20% strain. PCL reinforcement markedly improved $\sigma_{Eq}$, particularly in the circumferential and 3D configurations. Circumferential PCL reinforcement increased equilibrium stress to 3.04 ± 0.66 kPa at a 5% strain and 17.07 ± 5.77 kPa at a 20% strain, while 3D PCL scaffolds achieved even higher values, reaching 3.34 ± 0.41 kPa and 22.27 ± 7.56 kPa at 5% and 20% strains, respectively. In the 100 GTA hydrogels, $\sigma_{Eq}$ values were significantly higher across all configurations. Unreinforced hydrogels reached 1.52 ± 0.16 kPa at a 5% strain and 8.21 ± 1.00 kPa at a 20% strain, while circumferential PCL reinforcement elevated $\sigma_{Eq}$ to 3.45 ± 0.73 kPa at a 5% strain and 21.78 ± 3.57 kPa at a 20% strain. The 3D PCL configuration achieved the highest equilibrium stress values in the 100 GTA samples, with $\sigma_{Eq}$ reaching 4.14 ± 0.13 kPa and 25.03 ± 3.83 kPa at 5% and 20% strains, respectively. Statistical analysis confirmed significant effects of both strain level and PCL configuration for both GelA–GTA mass ratios on equilibrium stress (*p* < 0.0001). Post hoc Tukey’s tests indicated that the circumferential and 3D PCL configurations significantly outperformed the aligned and unreinforced hydrogels (*p* < 0.05), with circumferential PCL yielding the highest sustained stress values overall. These findings highlight the role of circumferential and 3D PCL reinforcements, particularly in 100 GTA hydrogels, in enhancing equilibrium stress [26,42]. The ability to sustain higher stress levels over extended periods reflects the potential of these configurations to mimic the load-bearing behavior of native meniscal tissue, making them promising candidates for meniscal replacement applications [25,43,44,45].

### 2.4. Aggregate Modulus ($H_{A}$)

**Figure 4:**
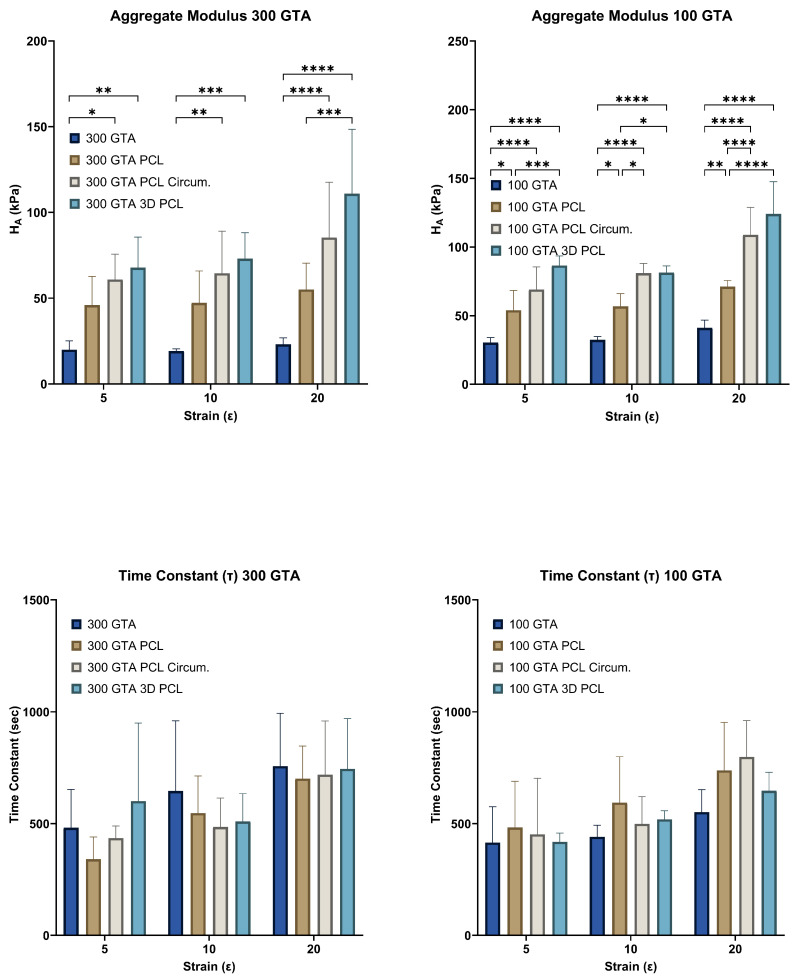
Aggregate modulus and time constant of HANI across different GelA–GTA mass ratios, PCL reinforcement types, and strain levels. Bar graphs show aggregate modulus ($H_{A}$) and time constant (τ) values for HANI scaffolds with varying GelA–GTA mass ratios (300 and 100) and PCL reinforcement types (no PCL, aligned PCL, circumferential PCL, and 3D PCL) at strain levels of 5%, 10%, and 20%. Higher aggregate modulus values are observed in hydrogels with circumferential and 3D PCL configurations, particularly in 100 GTA samples, indicating enhanced stiffness under increased strain. Time constant values vary with strain level, with no clear trend, reflecting diverse stress relaxation behavior across different configurations. Statistical significance is denoted by * *p* < 0.05; ** *p* < 0.01; *** *p* < 0.001; and **** *p* < 0.0001.

The aggregate modulus ($H_{A}$), representing the compressive stiffness under sustained load, increased significantly with higher strain levels and specific PCL reinforcement configurations for both GelA–GTA mass ratios (Figure 4). In the 300 GTA hydrogels, PCL reinforcement—particularly in the circumferential and 3D configurations—markedly improved $H_{A}$ compared to unreinforced samples, with the 3D configuration achieving the highest values. Similarly, the 100 GTA hydrogels, due to a greater cross-linking density, exhibited consistently higher stiffness across all configurations. The highest $H_{A}$ values were observed in the 100 GTA hydrogels with 3D PCL reinforcement, indicating superior compressive resistance. For both GelA–GTA mass ratios, the strain level and PCL reinforcement substantially enhanced the aggregate modulus. Circumferential and 3D PCL reinforcements provided the greatest stiffness improvements, particularly in the 100 GTA hydrogels, where the combination of a high cross-linking density and effective load distribution maximized compressive resistance. These results highlight the potential of these configurations for load-bearing applications like meniscal replacements, where sustained stiffness and structural integrity are crucial. The increased stiffness in the 100 GTA hydrogels with circumferential and 3D PCL reinforcement closely mirrors the compressive properties of native human meniscal tissue, with aggregate modulus values ranging in the order of 100–150 kPa [3,11,26,31,38]. These configurations demonstrate the durability and resilience required for long-term orthopaedic applications, making them strong candidates for meniscal tissue engineering. HANI’s combination of mechanical robustness and sustained compressive stiffness ensures both immediate load support and enduring performance under cyclic loading conditions [37,46,47].

### 2.5. Time Constant (τ)

The time constant (τ), indicative of the time required for hydrogels to reach equilibrium stress, exhibited minimal variation across PCL reinforcement types, suggesting limited influence on viscoelastic relaxation properties (Figure 4). In the 300 GTA hydrogels, τ values remained consistent across configurations, with circumferential and 3D PCL reinforcement showing slight decreases compared to unreinforced samples. Similarly, in the 100 GTA hydrogels, τ values were slightly higher for circumferential and aligned PCL reinforcements but did not show substantial changes [34]. The consistent τ values across strain levels and PCL configurations indicate that PCL reinforcement enhanced mechanical strength without significantly altering the gelatin matrix’s intrinsic relaxation dynamics for both GelA–GTA mass ratios. Reinforcement primarily influenced mechanical parameters like peak and equilibrium stress, while preserving viscoelastic stability [34,42,48]. The negligible effect of PCL reinforcement on τ suggests that the hydrogels retained their natural damping properties essential for energy dissipation and shock absorption [11,13]. These properties, combined with the mechanical benefits of PCL reinforcement, make hydrogels with circumferential and 3D PCL in a 100 GTA matrix particularly suitable for load-bearing applications like meniscal replacements.

### 2.6. Peak Modulus ($E_{Peak}$)

**Figure 5:**
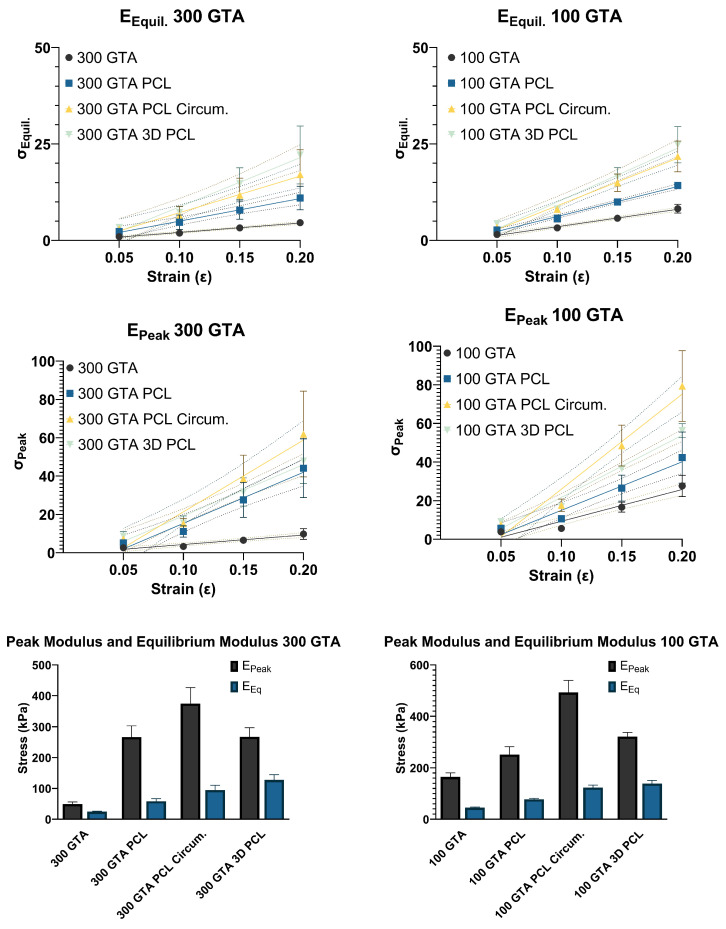
Peak and equilibrium modulus analysis of HANI with varying GelA–GTA mass ratios, PCL reinforcement types, and strain levels. The line graphs in the top panels show peak and equilibrium moduli for HANI formulated with 300 and 100 GelA–GTA mass ratios, with different PCL reinforcement types (no PCL, aligned PCL, circumferential PCL, and 3D PCL). The slopes of these lines correspond to the peak modulus (E_Peak_) and equilibrium modulus (E_Eq_). The bar graphs in the lower panels summarize the peak and equilibrium modulus values across the PCL configurations for each GelA–GTA mass ratio. Increased modulus values are noted in hydrogels with circumferential and 3D PCL configurations, particularly in the 100 GTA group, indicating superior mechanical reinforcement and resistance to deformation under strain. Error bars represent standard deviations.

The peak modulus ($E_{Peak}$), representing initial stiffness, increased significantly for both GelA–GTA mass ratios across PCL reinforcements (Figure 5). In the 300 GTA hydrogels, unreinforced samples showed a peak modulus of 49.58 kPa, which improved with aligned PCL (266.60 kPa), circumferential PCL (374.60 kPa), and 3D PCL (267.60 kPa). For the 100 GTA hydrogels, the unreinforced modulus of 164.80 kPa increased to 251.60 kPa with aligned PCL and peaked at 492.80 kPa with circumferential PCL. The 3D PCL configuration achieved 322.20 kPa, with a strong model fit (R^2^ = 0.96). Linear regression confirmed significant effects for both GelA–GTA mass ratios across PCL reinforcements on the peak modulus with all configurations having strong model fits (R^2^ > 0.745; *p* < 0.0001). The marked improvements in the peak modulus with circumferential and 3D PCL configurations highlight their ability to distribute compressive forces effectively, especially in 100 GTA hydrogels. These enhancements align with the mechanical demands of meniscal tissue engineering, where high initial stiffness is critical for load-bearing applications [49,50,51].

### 2.7. Equilibrium Modulus ($E_{Equil}$)

The equilibrium modulus ($E_{Equil}$), indicative of stiffness under sustained loads, showed substantial increases for both GelA–GTA mass ratios with PCL reinforcement (Figure 5). For the 300 GTA hydrogels, the unreinforced modulus of 24.43 kPa rose to 58.59 kPa with aligned PCL, 94.79 kPa with circumferential PCL, and 127.60 kPa with 3D PCL. In the 100 GTA hydrogels, circumferential PCL reached 123.60 kPa, while the highest value, 139.50 kPa, was recorded with 3D PCL. Linear regression confirmed significant effects for both GelA–GTA mass ratios across PCL reinforcements on the equilibrium modulus with all configurations having strong model fits (R^2^ > 0.735; *p* < 0.0001) with the exception of the 300 GTA circumferential PCL (R^2^ > 0.672; *p* < 0.0001). The high equilibrium modulus values achieved with circumferential and 3D PCL reinforcements in the 100 GTA hydrogels highlight their ability to sustain loads effectively over time. These configurations mirror the sustained stiffness of native meniscal tissue, demonstrating strong potential for use in durable, load-bearing meniscal replacements [25,50,52,53].

### 2.8. Optimizing Dimensional Stability in 3D PCL

**Figure 6:**
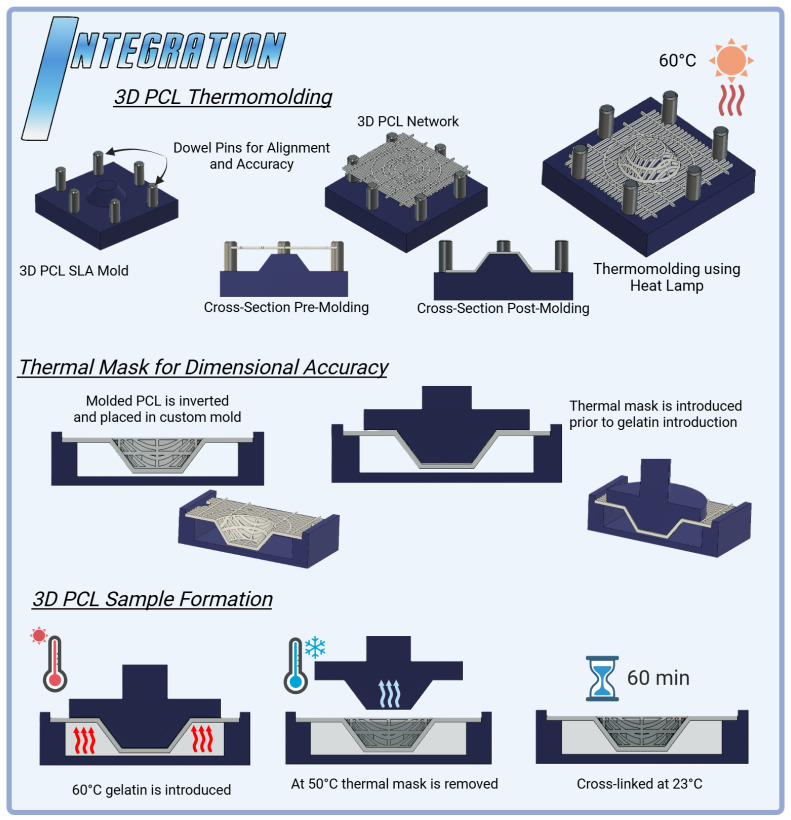
Integration techniques for HANI scaffolds: 3D PCL Thermomolding utilizes SLA-printed molds to shape PCL networks with circumferential bands into a spring-like 3D structure, achieved through controlled heat application at 60 °C. The process ensures precise alignment and enhanced mechanical properties. The 3D PCL thermal mask stabilizes the scaffold during curing, preventing deformation and maintaining dimensional accuracy. These integration methods optimize the structural integrity and mechanical performance of the HANI biomaterial, enabling a more effective replication of native meniscal properties.

Dimensional accuracy challenges in the 3D PCL configuration, primarily due to fiber elongation during thermomolding, could be addressed through strategic enhancements in mold design (Figure 6). The inclusion of dowel pins could improve fiber alignment and stability during molding, reducing misalignment and ensuring uniform network thickness [37,54,55,56]. Additionally, tailoring network patterns and thicknesses to specific mold geometries could help mitigate the effects of thermal expansion and elongation [57,58]. These refinements would enable precise control over fiber orientation, minimizing dimensional inaccuracies while promoting consistent compressive stability. Although this HANI study used a standardized PCL network thickness of 0.6 mm to maintain uniform polymer concentration, future mold-based modifications such as dowel pins and network customization hold promise for achieving greater dimensional precision and enhanced mechanical resilience. These advancements would yield more robust 3D PCL reinforcement, better suited to meeting the mechanical demands of load-bearing applications like meniscal tissue engineering [47,57,59].

### 2.9. Enhancing Dimensional Control with 3D Thermal Masks

A 3D thermal mask was employed to stabilize the PCL network during gelation, applying localized heat to preserve dimensional integrity and prevent fiber elongation. Post-cooling, the thermal mask was removed, ensuring that the network maintained its alignment and structural consistency. Further optimization of this process could enhance its efficacy (Figure 6). Fine-tuning the temperature gradient of the mask could target regions most prone to deformation, while adjustments to the timing and rate of mask removal may improve the retention of the intended shape, particularly where PCL fibers are embedded in gelatin [55,60,61]. These refinements could significantly enhance dimensional accuracy, ensuring consistent fiber alignment and improving mechanical stability. Such improvements would bolster the suitability of 3D PCL-reinforced hydrogels for load-bearing tissue engineering applications [9].

### 2.10. Design Implications

The HANI’s scaffold design leveraged advanced additive manufacturing techniques, combining Stereolithography (SLA) and Fused Deposition Modelling (FDM). SLA was pivotal in producing precise molds that preserved the alignment and stability of the PCL reinforcements, enabling networks to span the scaffold’s thickness and mimic the anisotropic properties of the native meniscus [21,57,62,63]. FDM enabled continuous fiber printing, creating circumferential and radial tie fibers that transformed axial compressive loads into tensile stresses. This reinforcement strategy, particularly in the 3D PCL configuration, ensured progressive load sharing and minimized localized deformation. Together, these techniques produced scaffolds capable of mimicking the hoop stress–strain behavior of the native meniscus, absorbing and redistributing forces effectively to protect surrounding cartilage [21,37,64,65].

### 2.11. Challenges and Limitations

It is important to note that variability in PCL placement can occur as a result of molding and fiber misalignment during hydrogel integration, potentially affecting mechanical properties and construct reproducibility. Future developments in the HANI process will focus on optimizing these fabrication techniques to ensure consistent PCL distribution, including the improvement of mold designs and the introduction of thermal stabilization strategies. Additionally, standardizing fabrication protocols and implementing more precise quality control measures, such as real-time imaging during scaffold formation, may help mitigate these inconsistencies in future studies. While the current scaffold fabrication approach produces reinforced hydrogel constructs with improved short-term mechanical performance, long term degradation and in vivo stability remain unexplored. Future research will focus on assessing scaffold behavior under physiological conditions in both in vitro and in vivo settings. These studies will be essential to validate the scaffold’s durability and clinical applicability for meniscal tissue engineering.

## 3. Conclusions

In this study, a novel scaffold fabrication approach (i.e., HANI) was introduced to develop gelatin-based hydrogel constructs that were reinforced with poly-ε-caprolactone (PCL) additive networks. Fused Deposition Modeling (FDM) was used to create PCL patterns that mimicked the structural organization of the native meniscus. PCL network integration was achieved using custom Stereolithography (SLA)-printed molds and thermal masks to ensure precise dimensional accuracy and seamless incorporation into the hydrogel matrix. Gelatin hydrogels reinforced with circumferential or 3D PCL networks showed notable improvements in peak, equilibrium, and aggregate modulus values. The time constant values remained stable across reinforcement configurations, indicating that PCL network integration enhanced structural integrity without altering the gelatin matrix’s intrinsic poroviscoelastic properties.

In conclusion, hydrogel composites reinforced with circumferential or 3D PCL networks emerge as potential candidates for meniscal implants, demonstrating high stiffness, sustained load tolerance, and resilience under repetitive compressive loading. In future studies, we plan to evaluate the biological performance of these constructs as a function of fabrication conditions (i.e., degree of cross-linking, PCL network configurations) in in vitro and in vivo settings.

## 4. Materials and Methods

The experimental design for this study was guided by a stepwise approach aimed at optimizing the mechanical and biological properties of HANI scaffolds for orthopedic soft tissue engineering applications. The HANI fabrication process began with hydrogels alone, fabricated using gelatin cross-linked with glutaraldehyde (GTA) to establish a baseline for their mechanical and viscoelastic performance. Gelatin hydrogels were selected for their excellent biocompatibility, high water content, and intrinsic poroviscoelastic properties, which are crucial for replicating the dynamic environment of the native meniscus [10,18,36,66].

However, hydrogels alone lack the mechanical strength necessary to endure the compressive and tensile forces experienced in the knee joint [11,12,51,67]. To address this limitation, HANI progressively integrate polycaprolactone (PCL) networks into the hydrogel matrix. These networks were fabricated using Fused Deposition Modeling (FDM) to introduce anisotropic, biomimetic patterns that mimic the structural organization of the meniscus [56]. By starting with unreinforced hydrogels (under two different cross-linking conditions) and subsequently incorporating PCL networks, the contribution of each PCL configuration to the mechanical properties of the hydrogel composites was evaluated.

The next phase involved refining the PCL network design by adding circumferential fibers and varying network configurations to enhance the scaffold’s anisotropy and load-distribution capabilities. Finally, 3D PCL networks were introduced to better control the strain range at which the PCL reinforcement engaged while maintaining the axial-to-tensile load conversion critical for meniscal function [3,4,5]. The 3D networks, designed with circumferential fibers spanning the scaffold’s thickness, ensured progressive activation under lower strains, further improving compressive stiffness and dynamic behavior [6,12,20,65,68].

This systematic approach reflected the thought process underlying the experiment: beginning with the simplest configuration to establish a baseline and incrementally adding complexity to optimize HANI’s mechanical, dynamic, and biological characteristics. With the aim of addressing the demanding requirements of orthopedic soft tissue such as the menisci, advanced and biomimetic scaffolds can be developed through iterative analysis processes followed by continuous design improvements.

### 4.1. Hydrogel Preparation

Gelatin hydrogels were prepared according to techniques described in our previous publication [69]. Briefly, GelA (gelatin type A from Porcine skin, 300 bloom, Sigma-Aldrich, St. Louis, MO, USA) was first dissolved in PBS (phosphate-buffered saline) to produce gelatin solutions at 0.15 g/ml concentrations. The gelatin solutions were heated in a water bath to 65 °C to achieve complete solubilization. Following solubilization, the gelatin solutions were added to molds along with various concentrations of glutaraldehyde (GTA) solution and immediately stirred. Hydrogels were then cross-linked for one hour and immersed in DI water for five minutes to arrest cross-linking (Figure 2).

### 4.2. Water Content at Various Mass Ratios

Glutaraldehyde (GTA) solutions were prepared at mass ratios of 25:1, 50:1, 100:1, and 300:1 by diluting stock 25% GTA solution (Sigma-Aldrich, Grade II, 25% in H_2_O) with PBS. A 2.5 mL aliquot of 15% GelA solution, prepared as previously described, was dispensed into individual molds with 25 mm diameters. The corresponding GTA solution was then promptly added and thoroughly mixed using a micropipette tip to ensure uniform cross-linking. Each mass ratio (25:1, 50:1, 100:1, 300:1) was tested using a sample size of *n* = 3. Following cross-linking, hydrogels were then transferred to weigh boats and left uncovered to desiccate under a fume hood for 24 h, ensuring complete dehydration (Figure 2).

### 4.3. Swelling Equilibration Point Determination

To assess the water content of the hydrogel samples, completely dehydrated specimens were initially weighed, and their dry weights were recorded. Subsequently, each sample was immersed in individual wells containing 5 mL of PBS solution. At predetermined time intervals (1, 2, 3, 4, 5, 6, 12, and 24 h), the samples were removed from the solution, gently patted dry to remove excess solution, and promptly weighed. All hydrogel groups were determined to have reached full osmotic equilibrium after a period of 12 h. The water content of the hydrogels was calculated using the following equation, where $Wt._{Wet}$ is the weight at osmotic equilibrium following swelling and $Wt._{Dry}$ is the weight following complete dehydration:

(1) $$\phi_{WC}=\frac{{Wt.}_{Wet}-{Wt.}_{Dry}}{{Wt.}_{Wet}}\times 100\%$$

### 4.4. Printing PCL Networks Using FDM

**Figure 7:**
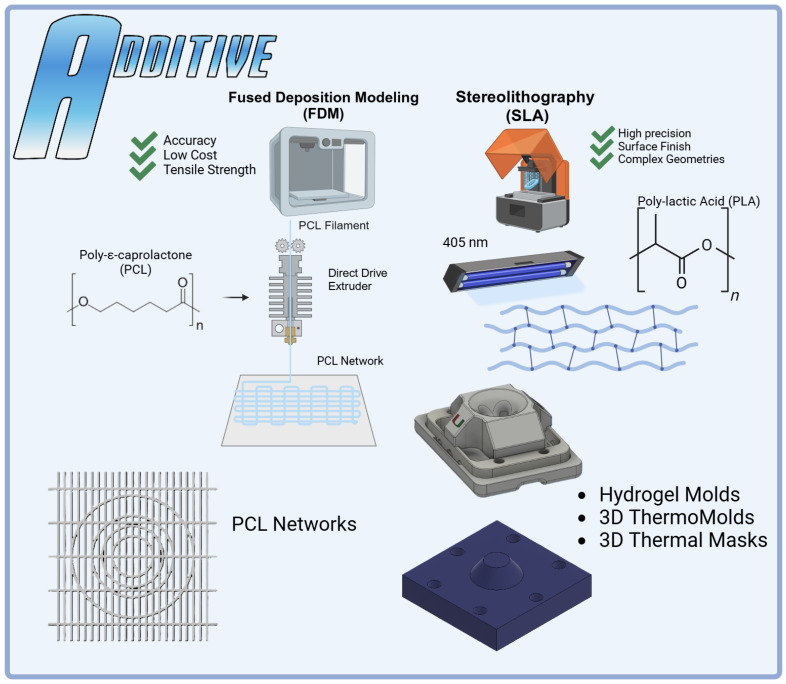
Additive manufacturing in the HANI scaffold process: Fused Deposition Modeling (FDM) was employed to create precise PCL networks using PCL filament and a modified Ender 3 printer, optimizing tensile strength and dimensional accuracy. Stereolithography (SLA) was utilized to fabricate complex hydrogel molds with high precision using PLA BioResin. The integration of both methods allowed the production of intricate 3D ThermoMolds and thermal masks, ensuring accurate alignment and stability during scaffold formation. Each technique played a specific role in enhancing the structural and mechanical properties of the HANI biomaterial.

**Figure 8:**
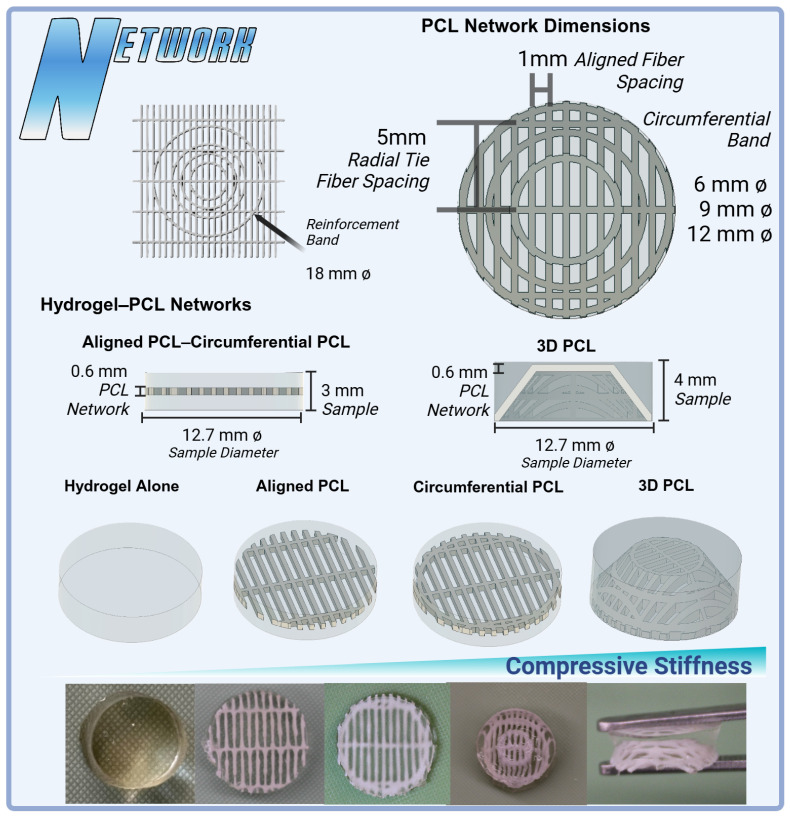
Variations in PCL network designs and their integration into hydrogel scaffolds for meniscal tissue engineering. Top: PCL network dimensions showcasing aligned fibers (1 mm spacing), radial tie fibers (5 mm spacing), and circumferential bands in configurations of 6 mm, 9 mm, and 12 mm diameters. Middle: Hydrogel–PCL scaffolds comparing hydrogel alone, aligned PCL, circumferential PCL, and 3D PCL constructs (4 mm sample height). The 3D PCL structure demonstrates enhanced anisotropy and load-bearing potential. Bottom: A visual comparison of fabricated constructs, illustrating the transition from hydrogel-only samples to PCL-reinforced designs, highlighting improvements in compressive stiffness with additional layers and network complexity.

Printed polycaprolactone (PCL Filament 1.75 mm 50,000 g/mol MW) networks were fabricated using FDM on a modified direct-drive Ender 3 printer (Figure 7). A PCL network with a thickness of 0.6 mm was printed utilizing a 0.2 mm layer height and 0.4 mm nozzle size with aligned fibers spaced 1mm apart and orthogonal radial-tie fibers sapced at 5 mm intervals. A second PCL network was then printed with the addition of a secondary circumferential fiber of a 12 mm diameter to further connect the aligned fibers of the PCL printed structure, mimicking the fibers’ ability to transform axial loads into tensile stress and improve compressive stiffness like native menisci [17]. Then, additional circumferential bands were added at diameters of 6 mm and 9 mm for the 3D thermomolding process (Figure 8). All prints were produced with a nozzle temperature of 141 °C, a bed temperature of 33 °C, and a print speed of 22 mm/s.

### 4.5. Integration

HANI were prepared by integrating a PCL network into the existing hydrogel preparation via the use of a custom mold fabricated via SLA [56]. PCL networks were placed in PBS and refrigerated to 4 °C and then immediately removed and blotted dry prior to the introduction of gelatin into the mold to mitigate warping due to thermal effects from the gelatin and maintain dimensional accuracy. HANI process molds (Figure 6) were designed to ensure that the PCL networks maintained dimensional accuracy and a proper position within the hydrogel matrix and to mitigate warping during gelatin introduction [37]. SLA thermomolds and thermal masks were used during the production of 3D PCL samples to help ensure the dimensional accuracy and retention of the molded 3D shapes. The thermal masks were removed once the gelatin had cooled to below 55 °C.

### 4.6. Three-Dimensional PCL Network Thermomolding

To further investigate the effects of controlling the mechanical properties of integrated PCL reinforcement networks, aligned PCL networks with circumferential bands were processed into 3D springs using custom SLA molds. The objective was to improve the overall compressive behavior of the HANI biomaterials across various strain levels while maintaining poroviscoelastic stress relaxation behavior. Initially, the composite structure of the hydrogel had the PCL network embedded centrally. However, the 3D PCL configuration ensured that the PCL fibers spanned the entire thickness of the sample. This design aimed to engage the PCL reinforcement at lower strain levels (Figure 8), thereby further mimicking the native meniscus’ circumferentially aligned collagen bundles in the deep layer [27].

### 4.7. Confined Compression Stress Relaxation Testing

**Figure 9:**
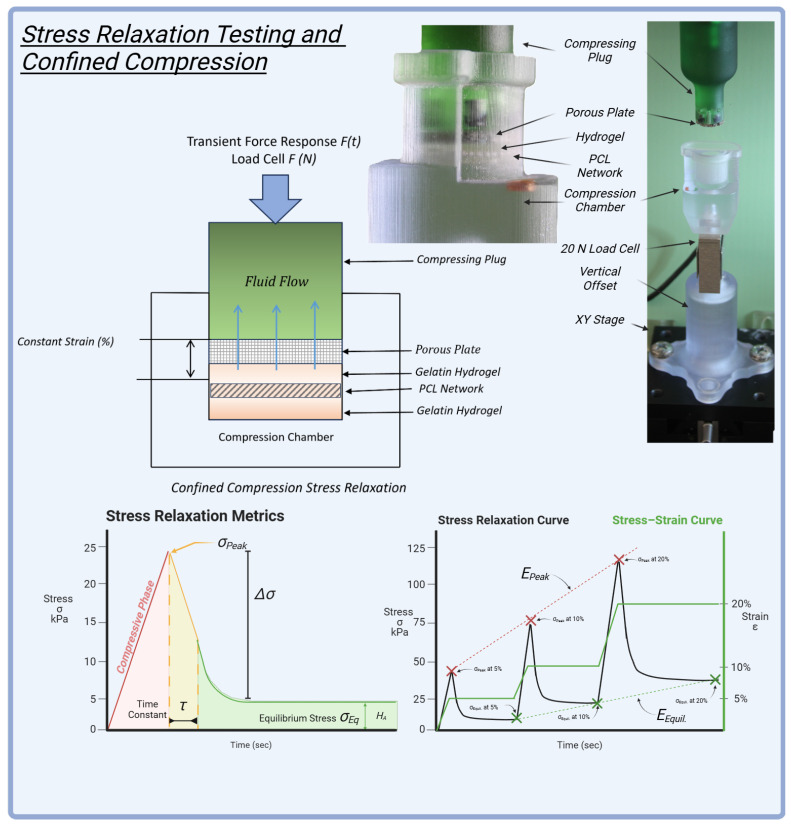
Stress relaxation testing and confined compression: The setup featured a custom confined compression chamber with a porous plate, hydrogel, and an embedded PCL network, designed for precise mechanical evaluation. A 20 N load cell measured the transient force response during compression. The stress relaxation metrics included peak stress ($\sigma_{Peak}$), equilibrium stress ($\sigma_{\mathrm{Eq}}$), and the time constant (τ), indicating the material’s viscoelastic behavior. The stress–strain curve illustrates the response at 5%, 10%, and 20% strain levels, providing insights into compressive stiffness and relaxation properties. This testing protocol helped assess the mechanical performance of the HANI, replicating the dynamic behavior of native meniscal tissue.

HANI were evaluated using a confined compression stress relaxation protocol to assess their mechanical properties and viscoelastic behaviour under various loading conditions (Figure 9). These tests were conducted in a custom-built confined compression chamber [69]. The hydrogels were examined in both PCL-reinforced and unreinforced forms, with GelA–GTA mass ratios of 300 and 100, generating six HANI configurations and two hydrogel-only controls.

### 4.8. Experimental Protocol for Stress Relaxation

The stress relaxation protocol for HANI involved compressing each sample progressively to different strain levels. After a preload of 0.15 N for 1 min, samples were compressed at a rate of 3.12 µm/s to an initial strain of 5% and held for 30 min, followed by compression to 10% strain for another 30 min and finally to 20% strain, held for 60 min. This stepwise approach ensured that key mechanical parameters, such as peak and equilibrium stresses, were captured at multiple intervals (Figure 9), reflecting the hydrogels’ capacity to handle sustained physiological loads across physiologically relevant strains.

### 4.9. Stress Relaxation Behavior and Curve Fitting

To further characterize the poroviscoelastic properties, stress relaxation data from each strain level were fitted to a single-phase logarithmic decay model in Python 3.9 using the following stress decay equation:

(2) $$\sigma \left(t\right)=\Delta \sigma e^{-t/\tau }+\sigma_{Eq}$$

where $\sigma_{0}$ represents the peak stress at the start of the constant strain hold immediately following compression (*t* = 0), Δσ is the initial decay stress equal to the difference between initial peak stress and equilibrium stress, τ is the relaxation time constant, and $\sigma_{Eq}$ denotes the equilibrium stress measured at the end of each hold phase. The relaxation time constant τ quantifies how quickly the stress dissipates under constant strain, reflecting the scaffold’s ability to redistribute mechanical forces, an essential feature for load-bearing tissues.

### 4.10. Peak Modulus

The peak modulus $\left(E_{Peak}\right)$ quantifies the material’s instantaneous stiffness during the initial loading phases. To determine the peak modulus, the peak stresses at different strain levels (ε = 5%, 10%, 15% (average), and 20%) are plotted against the corresponding strains, and the slope of the linear regression line represents the peak modulus:

(3) $$E_{Peak}=\frac{\Delta \sigma_{0}}{\Delta \varepsilon }$$

This slope reflects the scaffold’s resistance to deformation under compressive loads, with a higher slope indicating stiffer hydrogels. The peak modulus is essential for evaluating whether the HANI provide the necessary mechanical integrity to handle dynamic joint stresses.

### 4.11. Equilibrium Modulus

The equilibrium modulus ($E_{Eq}$) measures the stiffness of the material at equilibrium, once internal fluid flow has ceased. This modulus is particularly relevant for biological tissues such as cartilage and menisci, where maintaining structural integrity under sustained loads is crucial for long-term functionality [70,71]. During stress relaxation testing, equilibrium stresses, $\sigma_{Eq}$, are recorded at the end of each hold phase. To determine the equilibrium modulus, equilibrium stresses at different strain levels (ε = 5%, 10%, 15% (average), and 20%) are plotted against the corresponding strains, and the slope of the linear regression line represents the equilibrium modulus:

(4) $$E_{Equil}=\frac{\Delta \sigma_{Eq}}{\Delta \varepsilon }$$

### 4.12. Aggregate Modulus

The aggregate modulus, $H_{A}$, is a key measure of a material’s stiffness under compressive loading, particularly relevant in evaluating hydrogels and biological tissues like cartilage. It represents the equilibrium stress $\sigma_{Eq}$ divided by the applied strain ε, characterizing the material’s resistance to deformation when internal fluid flow has equilibrated. In tissue engineering, a higher aggregate modulus indicates greater compressive stiffness, essential for materials designed to support mechanical loads in joint or load-bearing applications [26,38,45].

(5) $$H_{A}=\frac{\sigma_{Eq}}{\varepsilon }$$

### 4.13. Statistical Analysis

Statistical analyses were conducted using GraphPad Prism 10.3 (GraphPad Software, San Diego, CA, USA) to evaluate the effects of PCL reinforcement configurations (aligned, circumferential, and 3D PCL) and strain levels (5%, 10%, and 20%) on the mechanical properties of two distinct gelatin hydrogels: (a) those cross-linked at a 300:1 GelA-to-GTA ratio (lower degree of cross-linking) and (b) those cross-linked at a 100:1 GelA-to-GTA ratio (higher degree of cross-linking). Mechanical properties that were analyzed included peak stress, the stress delta, equilibrium stress, the time constant, the aggregate modulus, and the corresponding modulus values. Two-way ANOVA was performed to examine the effects of PCL configuration and strain level on these mechanical properties.

Tukey’s multiple-comparison test was used post hoc to conduct pairwise comparisons across combinations of PCL reinforcement types and strain levels for each hydrogel composition. Linear regression analysis was applied to determine peak and equilibrium moduli by calculating the slope of peak and equilibrium stress across strain levels. An α = 0.05 value was set for all tests, with a sample size of *n* = 5 for each group.

## Data Availability

The raw data supporting the conclusions of this article will be made available by the authors on request.

## Abbreviations

The following abbreviations are used in this manuscript:

GTA	Glutaraldehyde
PCL	Polycaprolactone
GelA	Gelatin A
FDM	Fused Deposition Modeling
SLA	Stereolithography
